# Radiation risk and mammographic screening of women from 40 to 49 years of age: effect on breast cancer rates and years of life

**DOI:** 10.1054/bjoc.1999.0903

**Published:** 1999-12-08

**Authors:** A Mattsson, W Leitz, L E Rutqvist

**Affiliations:** Oncologic Centre M8:01, Karolinska University Hospital, Stockholm, S-171 76, Sweden; Swedish Radiation Protection Institute, Stockholm, S-171 16, Sweden

**Keywords:** mammographic screening, radiation risk, life years

## Abstract

The aim of this study was to evaluate the carcinogenic risks associated with radiation in mass mammographic screening. Assessment was in terms of breast cancer mortality and years of life for a hypothetical cohort of 100 000 women. Data were obtained on incidence, mortality and life expectancy for the female population of Stockholm. With a screening interval of 18 months at ages 40–49 years, a total absorbed dose to the breast of 13 mGy per invited woman; and an annual breast cancer reduction of 25% per year 7 years from screening start, the net number of years gained was at least 2800. However, using the highest absorbed dose reported in routine mammographic screening in Sweden (≈3 mGy per view), and the highest reported radiation risk in the literature, a programme entailing annual screening with 2 views would require at least a 20% annual reduction in breast cancer mortality to give a net benefit in both the number of years of life gained and number of breast cancer deaths avoided. This observation supports the conclusion that exposures with low absorbed dose are essential when performing mass screening with mammography among young women. © 2000 Cancer Research Campaign


*British Journal of Cancer* (2000) **82**(1), 220–226
© 2000 Cancer Research Campaign
Article no. bjoc.1999.0903

### Radiation risk and mammographic screening of women from 40 to 49 years of age: effect on breast cancer rates and years of life

**A Mattsson[1], W Leitz[2] and LE Rutqvist[1]**

[1]Oncologic Centre, M8:01, Karolinska University Hospital, S-171 76 Stockholm, Sweden; [2]Swedish Radiation Protection Institute, S-171 16 Stockholm, Sweden

**Summary** The aim of this study was to evaluate the carcinogenic risks associated with radiation in mass mammographic screening. Assessment was in terms of breast cancer mortality and years of life for a hypothetical cohort of 100 000 women. Data were obtained on incidence, mortality and life expectancy for the female population of Stockholm. With a screening interval of 18 months at ages 40–49 years, a total absorbed dose to the breast of 13 mGy per invited woman; and an annual breast cancer reduction of 25% per year 7 years from screening start, the net number of years gained was at least 2800. However, using the highest absorbed dose reported in routine mammographic screening in Sweden (≈3 mGy per view), and the highest reported radiation risk in the literature, a programme entailing annual screening with 2 views would require at least a 20% annual reduction in breast cancer mortality to give a net benefit in both the number of years of life gained and number of breast cancer deaths avoided. This observation supports the conclusion that exposures with low absorbed dose are essential when performing mass screening with mammography among young women. © 2000 Cancer Research Campaign

**Keywords:** mammographic screening; radiation risk; life years

Cumulative reduction in breast cancer mortality has been shown in a number of controlled mammographic screening studies (Shapiro et al, 1988; Fletcher et al, 1993; Nystrom et al, 1993; Gordis et al, 1997). Such reductions for women 50–69 years of age at screening have been observed to be about 30% 10–12 years after study entry, and for this age-group mammographic screening is today an accepted method. For women 40–49 years of age, however, most of the individual trials have so far not demonstrated statistically convincing evidence of reduction in breast cancer mortality, and the beneficial effect of breast cancer screening is still under debate. However, recent results from the Gothenburg and Malmö studies are indicating a statistically significant reduction of about 40% (95% confidence interval 5–70%) (Andersson and Janzon, 1997; Bjurstam et al, 1997).

Among adverse effects that should be taken into account is the risk for radiation-induced breast cancer, especially for women in the lower age group. In most published epidemiological studies this risk has been found to be higher for women < 50 years of age compared to women ≥ 50 years of age at time of exposure (UNSCEAR, 1988). For a given age at exposure the excess relative risk estimates per Gy found in the various studies differ within a factor of four (Mattsson et al, 1995).

The balance between benefit and risk is crucial for the outcome of a screening programme. In this paper we calculate measures of efficiency under varying assumptions for the reduction in breast cancer mortality and for the risk of radiation-induced breast cancer respectively. The calculations cover a long period of time because radiation-induced breast cancer deaths can occur after a relatively long latency. The main purpose of this study was to evaluate the effect of a mammographic screening programme on breast cancer incidence and mortality as well as the associated years of life gained or lost. This was achieved by following a hypothetical cohort of 40-year-old women throughout life.

*Received 5 January 1999*
*Revised 9 June 1999*
*Accepted 16 June 1999*

*Correspondence to:* A Mattsson

### MATERIALS AND METHODS

In different breast cancer screening trials the reduction in breast cancer mortality has been expressed in relative terms, which means that the higher the background breast cancer mortality, the higher the number of years of life gained for a given reduction. Breast cancer mortality is lower in Sweden compared to some other Western societies, such as The Netherlands, the UK or the US (Bjurstam et al, 1997). In this study we have chosen population-based data from the female population of Stockholm to calculate the effects on years of life. Compared to women in the UK these women have similar breast cancer incidence rates (IARC, 1992), but around 20% lower breast cancer mortality rates (Berrino et al, 1995; Stockholm Breast Cancer Study Group, 1997).

Measures of effect were related to background breast cancer mortality rates (i.e. rates that would persist if screening was not present) and based on a cohort of 100 000 40-year-old women with no history of breast cancer being followed until 100 years of age. During the follow-up, the size of the cohort decreased by death risks specific to the attained age. For details of calculations see Appendix.

### Background breast cancer mortality

The background breast cancer mortality was calculated using number of breast cancers diagnosed per year of age, cumulative survival rates and annual death rates. Incidence data were obtained

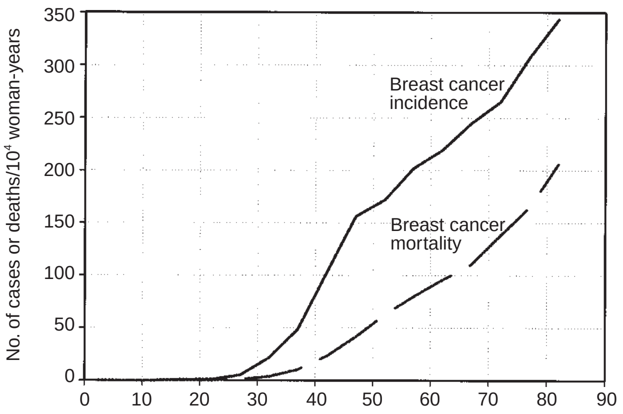

**Figure 1** Age-specific breast cancer incidence rate of first primary tumours per 100 000 women in Stockholm county during 1977–1986 and calculated age-specific breast cancer mortality rates

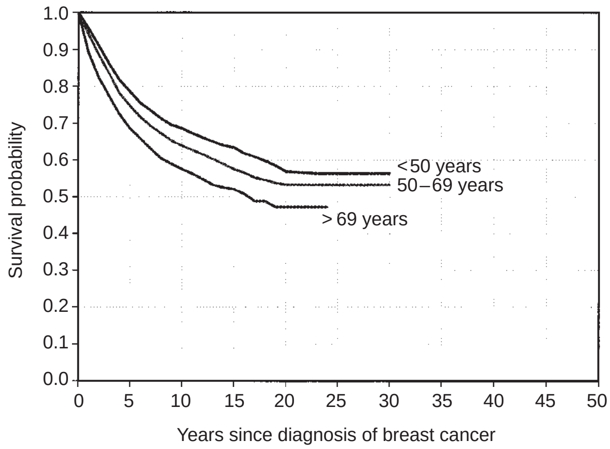

**Figure 2** Survival rates for women aged <50 years, 50–69 years, and >69 years at diagnosis of breast cancer in Stockholm county. For details see text

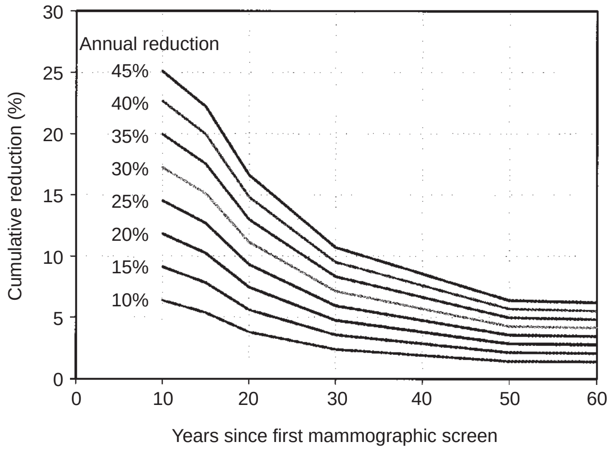

**Figure 3** Cumulative breast cancer mortality reduction for a group being screened at age 40–49 entailing different annual reduction from the Regional Cancer Register of the Stockholm County for period of diagnosis 1977–1986 (Figure 1). Survival and death rates for the first 20 years after diagnosis were obtained from patients diagnosed during 1970–1979, and the rates 20–29 years after diagnosis from patients diagnosed during 1961–1969. These data were thus from 1970–1988. This period was chosen to minimize the influence of mammographic screening programmes on background breast cancer mortality rates and to be as current as possible. Mammographic screening programmes were introduced in the Stockholm region in 1989.

The survival rates were confined to breast cancer deaths only and calculated using data from the Swedish Cause of Death Register for the patients registered in the Stockholm–Gotland Regional Cancer Register. No breast cancer death was reported among these patients more than 20 years after the initial diagnosis. The rates are shown graphically in Figure 2. The calculated breast cancer mortality rates are shown in Figure 1.

### Effects of mammographic screening

### *Reduction in background breast cancer mortality*

The effect of screening was calculated assuming a reduction of the background rate, i.e. of the breast cancer mortality generated among breast cancer patients diagnosed during selected ages with mammographic screening. Numerical values for the reduction are available from the overview of the Swedish randomized mammography screening trials (Nystrom et al, 1993; Tabar et al, 1996). In these trials, the effect was not seen until a certain time period after the first screening round. For women 40–49 years of age at screening, this lag-period was 7 years and for women between 50 and 69 years of age it was 3 years (Nystrom et al, 1993).

For the time after the lag-period we assumed a constant annual reduction of the breast cancer mortality (‘P’ in Appendix). This might be a too large simplification, but is not unrealistic given the Swedish overview results (Nystrom et al, 1993). For ages between 40 and 49 years at screening we made calculations for an annual breast cancer mortality reduction in the range 10–45%. This range corresponds to a cumulative reduction of 7–25% 10 years after screening start (given no screening after 50 years of age; Figure 3). In the overview of the Swedish randomized trials, the cumulative reduction was 13% after a median follow-up of 9 years. The attendance rate was ≈80% (Nystrom et al, 1993). In a recent update the cumulative reduction was reported to 23% after a median follow-up of 13 years (Tabar et al, 1996). In the 11-year follow-up of the Gothenburg breast screening trial a 45% cumulative reduction was observed (Andersson and Janzon, 1997). However, due to the design of these studies, it is difficult to quantify the part of the cumulative reduction that relates to screening before 50 years of age (Gordis et al, 1997).

For the ages at screening between 50 and 69 years, we used an annual reduction of 30%. This corresponds to a cumulative reduction of approximately 29% after 12 years, which thus reflects the results of the Swedish overview (Nystrom et al, 1993). For comparative purposes analyses were also done using a 20% and a 40% annual reduction respectively. When appropriate, the annual reductions are referred to as ‘x% reductions’.

The mean lead time introduced by screening has been estimated at 3 years (Rimer, 1996), which implies that the breast cancer incidence is lower than the background after a screening programme is stopped. We assumed the incidence to increase by

© 2000 Cancer Research Campaign

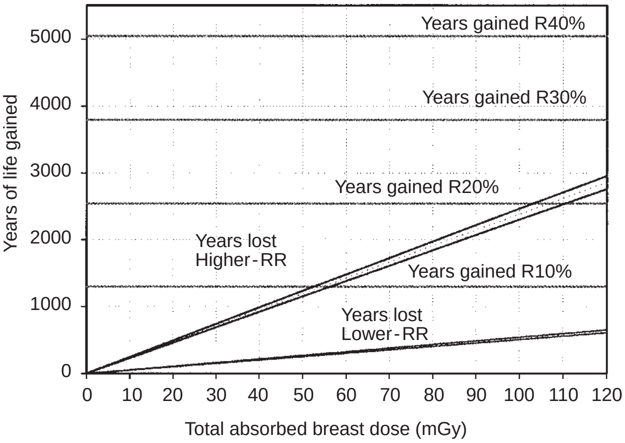

**Figure 4** Number of years of life gained and lost as a function of mortality reduction, radiation risk assumption and radiation dose. For details see text

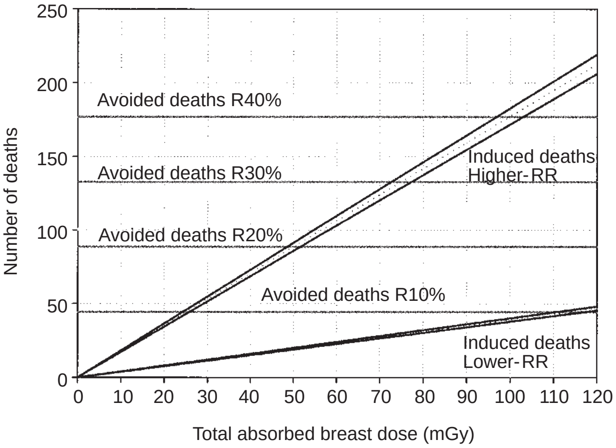

**Figure 5** Number of avoided breast cancer deaths and induced breast cancer deaths as a function of mortality reduction, radiation risk assumption and radiation dose. For details see text one-third of this difference per year after the screening has come to an end so that the background level is reached 3 years later.

### *Induced breast cancer cases*

When calculating the number of radiation-induced breast cancers due to a given mammographic screening programme involving a certain radiation dose, four factors are of importance: the excess relative risk (ERR) of breast cancer caused by ionizing radiation; the latency time for the excess to emerge; the background breast cancer incidence; and the decrease by attained age (time) of the size of the screened population.

Breast cancer risk. Results for women exposed to low doses are not available; in fact, it would be impossible to come to conclusive results with doses present in screening programmes (dose to the breast less than a few hundred mGy) even with hundreds of thousand of women in a study (Land, 1980). So, estimates of breast cancer risks from low-dose radiation exposures must be based on information obtained from studying populations exposed to much higher doses. Extrapolations to low doses are usually based on the use of theoretical and experimental radiobiological target theories, where the risk increases linearly at low doses with an upward curvature at medium dose levels (UNSCEAR, 1993).

We considered the results from the four most informative breast cancer risk studies, i.e. the A-bomb survivors study (Tokunaga et al, 1994), the Massachusetts fluoroscopy study (Boice et al, 1991), the New York mastitis study (Shore et al, 1986) and the benign breast disease study (Mattsson et al, 1995). In accord with these studies we assumed a linear increase in ERR with dose yet modified by age-at-exposure. (The higher age-at-exposure the lower ERR $Gy^{-1}$.) The excess incidence by attained age was taken relatively to the background incidence rates. We also assumed the excess risk to remain elevated throughout life (UNSCEAR, 1994). However, risk gradients differ between the studies. Because we cannot determine which is the most relevant we made calculations both with a high-risk and with a low-risk alternative. The high-risk alternative chosen was the ERR observed for women irradiated for benign breast disease (Mattsson et al, 1995) and the low-risk alternative chosen was from women with lung tuberculosis who received lung collapse therapy and who underwent fluoroscopy in Massachusetts (Boice et al, 1991). Formulas for the dose–response models are given in the Appendix.

Latency. We used a graded impact of the ERR that increases to full impact 10 years after exposure according to figures published by the National Institute of Health (NIH, 1985) (see Appendix).

Background rates. The same incidence rates as for the calculation of background mortality were used (Figure 1). These rates are not influenced by mammographic screening programmes introduced later.

Size of population. The hypothetical screening population decreased in size by death risks on a year by year basis. Data were obtained from the female population of Stockholm during 1980–1989 (Institute of Regional Analyses in Stockholm, INREGIA). Because women diagnosed with breast cancer and still alive are not invited to service screening, they too were excluded, year by year, from the hypothetical screening population.

### *Induced breast cancer deaths*

The number of induced breast cancer deaths was calculated in analogy to the calculation of the background breast cancer deaths. The number of registered breast cancers was replaced by the calculated number of induced breast cancers. The effect of the screening programme due to the reduction in breast cancer mortality on the number of induced deaths was calculated in the same way as for the number of breast cancer deaths described above and in the Appendix.

### *Net effects*

The net number of avoided breast cancer deaths was calculated as the difference between the background breast cancer mortality and the breast cancer mortality with mammography (with the induced breast cancer mortality taken into account) with summation from the starting age for the hypothetical cohort (i.e. 40 years) up to 100 years of age. In calculating the years of life gained (or lost), life table data for women in Stockholm county during the period 1986–1990 were used (INREGIA).

 © 2000 Cancer Research Campaign

**Table 1** Breast cancer incidence and mortality without and after mammographic screening of a hypothetical cohort of 100 000 women aged 40 years followed to 100 years of age[1]

| | Screening between 40–49 years of age | | 40–69 years of age | |
|---|---|---|---|---|
| | Radiation risk | | | |
| | Higher[2] | Lower[3] | Higher[2] | Lower[3] |
| Cumulative incidence | | | | |
| No mammography, % | 9.29 | 9.29 | 9.29 | 9.29 |
| + radiation-induced cases, % | 9.35 | 9.30 | 9.37 | 9.31 |
| No. of induced cases | 53 | 12 | 76 | 16 |
| Cumulative mortality | | | | |
| No mammography, % | 4.05 | 4.05 | 4.05 | 4.05 |
| With mammography[4], % | 3.96 | 3.94 | 3.40 | 3.38 |
| Percentage reduction | 2.1 | 2.6 | 15.9 | 16.5 |
| No. of avoided deaths | 111 | 111 | 674 | 674 |
| No. of induced deaths | 24 | 5 | 31 | 7 |
| Avoided/induced deaths | 5 | 21 | 22 | 104 |
| No. of gained years | 3170 | 3170 | 13 500 | 13 500 |
| No. of lost years | 325 | 71 | 357 | 75 |
| Avoided/induced years | 10 | 45 | 38 | 180 |

[1]Two programmes were compared: a. Screening from 40 to 49 years of age with in average 1.5 views at 1.5 mGy per examination every 18 months (total dose/woman ≈13 mGy). b. Same as 1: + biannual examinations 1.5 views at 1.5 mGy from 50 to 69 years of age (total dose/woman ≈33 mGy). Attendance rate 80%. Recall rate: 5% for <50 years of age; 3% for ≥50 years of age. Three views at recall examination. [2]ERR estimated for women in the benign breast disease study (Mattsson et al, 1995). [3]ERR estimated for women in the Massachusetts fluoroscopy study (Boice et al, 1991). [4]Annual reduction in breast cancer mortality was 25% beginning 7 years after first examination for the 40- to 49-year age-group. For the 50- to 69-year age-group a 30% annual reduction was used. The reduction assumed to stay throughout life.

### RESULTS

### Screening of women from 40 to 49 years of age

The number of years of life gained and lost and the number of deaths avoided and induced for our cohort of 100 000 women is shown in Figures 4 and 5 respectively. R10%, …, R40% denote a 10%, …, 40% reduction in breast cancer mortality respectively. ‘Higher-RR’ correspond to the radiation risk observed in the benign breast disease study (Mattsson et al, 1995). The effect is slightly dependent on the magnitude of the reduction of breast cancer mortality, which is indicated in the Figure. The dotted line is the result for a 20% reduction in breast cancer mortality, and the band around the dotted line represents the range between 40% and 0%. ‘Lower-RR’ corresponds to the risk observed in the Massachusetts fluoroscopy study (Boice et al, 1991).

The total number of years of life gained associated per percentage unit in reduction of breast cancer mortality was approximately 130 years. The number of years of life lost was around 5 per mGy for the lower and around 24 per mGy for the higher radiation risk assumption.

The dose for which the number of induced breast cancer deaths becomes equal to the number of avoided breast cancer deaths is lower than that for the correspondent comparison for the number of years of life (Figures 4 and 5). The reason is that, on average, radiation-induced breast cancer deaths occur later than the avoided breast cancer deaths would have had.

In the specific scenario for 40–49 years of age we assumed the screening interval to be 18 months as recommended by the health authority. Dose values from routine mammography in Sweden 1994 were used (W Leitz, unpublished data). Per screening round, 1.5 views were taken on average with a mean absorbed dose of 1.5 mGy per view. Assuming an attendance rate of 80%, a recall rate of 5% and three views at recall examination gives a total absorbed dose for the programme of ≈13 mGy per invited woman. The annual reduction in breast cancer mortality associated with the screening programme was assumed to be 25% from 7 years after first mammographic screen and remaining throughout life. The corresponding cumulative reduction is given in Figure 3.

The lifetime cumulative risk of breast cancer for such a programme increased from 9.29% to 9.30% or 9.35% with the lower and higher radiation risk assumption respectively (Table 1). The net number of years of life gained was between 2800 and 3100 (Table 1).

Delaying the screening start in steps of 1 year, from 40 to 41, 42, …, 49 years of age (and assuming a 25% annual breast cancer mortality reduction) gradually decreased the number of net gained years from a maximum of 3100 to 1200. The ratio between avoided and induced deaths increased from 5 to 21 for the higher radiation risk alternative and from 26 to 128 for the lower radiation risk assumption respectively. The ratio between gained and lost years was approximately 2 times higher.

For routine mammographic screening in Sweden 1994 the 90th percentile of the absorbed breast dose distribution was observed to 1.9 mGy per view and the corresponding number of views per examination to two (W Leitz, unpublished data). For these values (for screening interval of 18 months and 25% reduction in breast cancer mortality) net years gained were (with figures for the higher radiation risk assumption presented first) 2600:3100 (ratio avoided/induced deaths: 2.8: 12.7). Using the most extreme mean absorbed breast dose reported for mammographic screening in Sweden 1994 (3.2 mGy per view) reduced the net years gained to 2300: 3000 (ratio avoided/induced deaths: 1.7:7.6).

### Screening of women from 40 to 69 years of age

The same assumptions as in the first scenario (i.e. for 40- to 49-year-old women) were used. For the ages 50–69 years, we assumed that 1.5 views were taken (1.5 mGy per view) every second year from 50 years of age. Participation rate was 80% and recall rate 3% with three views at 1.5 mGy. The 2-year screening interval represents the mean interval for the Swedish randomized trials (Nystrom et al, 1993). The total cumulative absorbed dose for the programme was ≈33 mGy per invited woman.

We used a 30% annual reduction in breast cancer mortality for ages at screening of 50–69 years. Such an annual reduction would give a cumulative reduction 15 years from start of screening of ≈28%, in a population of 50-year-old women with no previous diagnosis of breast cancer. The cumulative figure is lower than the annual figure due to the 3-year lag-time for the screening benefit that was assumed. However, for our scenario with screening from 40 to 69 years of age the assumption of a 3-year lag-time from 50 to 52 years of age was changed from no effect to the same effect as used for the 40- to 49-year age-group.

Figure 6 shows the cumulative reduction of breast cancer mortality for different annual breast cancer mortality reductions as a consequence of screening at ages 40–49 years, and a given reduction of 30% of screening at ages 50–69 years.

*© 2000 Cancer Research Campaign*

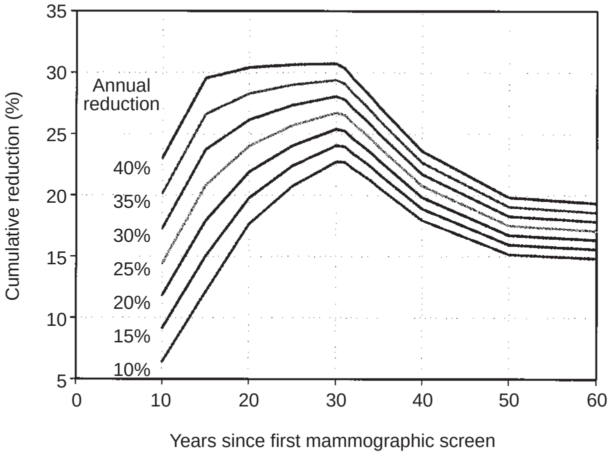

**Figure 6** Cumulative breast cancer mortality reduction for a group being screened at age 40–49 entailing different annual reductions, given a 30% annual reduction for subsequent screening between 50 and 69 years of age

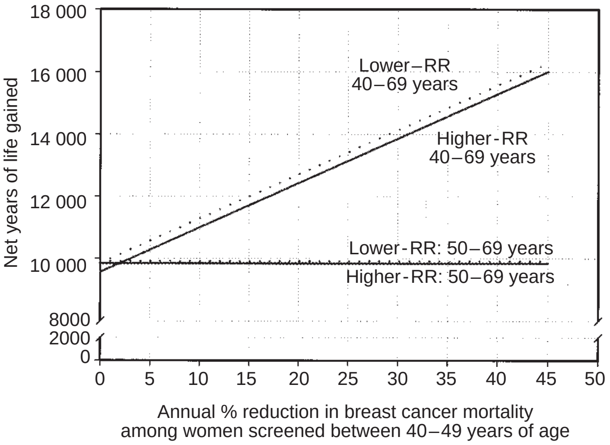

**Figure 7** Net gain of years of life after a screening programme between 40 and 69 years of age as a function of the screening effect for the 40- to 49-year age-group given a 30% annual reduction for the 50- to 69-year age-group

The 30% breast cancer mortality reduction associated with screening at ages 50–69 years resulted in a net gain of approximately 9900 years of life, independently of radiation risk assumption. Varying the assumption of the reduction figure by 10% changed the net gain of years by approximately 3300.

The additional numbers of gained years as a consequence of screening the 40- to 49-year age-group was 140 per percentage unit change in breast cancer mortality reduction given the 30% reduction for the older age group (Figure 7).

A 25% reduction for the 40 to 49 (i.e. 40–52)-year age group gave an additional benefit of around 3200 years of life for the higher radiation risk assumption and 3500 for the lower respectively (Figure 7). The lifetime cumulative risk of breast cancer due to the radiation exposure increased from 9.29 to 9.31 for the lower radiation risk level and to 9.37 for the higher respectively (Table 1). The effect on cumulative reduction in breast cancer mortality up to 100 years of age was around 16% (Table 1).

### DISCUSSION

In this study we focused on the trade-off on breast cancer mortality between avoided breast cancer deaths (due to earlier diagnosis) and induced breast cancer deaths (due to ionizing radiation) associated with different mammographic screening policies for a lifelong follow-up of a hypothetical cohort of 100 000 women.

There was a positive net effect in breast cancer mortality reduction for absorbed doses within reasonable limits for women between 50 and 69 years of age at screening and using figures for breast cancer mortality reductions as seen in the overview of the Swedish randomized mammography screening trials (Nystrom et al, 1993).

However, the main focus in this report was on mammographic screening of women aged 40–49 years. Generally, even in this age group the results indicate a net benefit in terms of years of life gained and breast cancer deaths avoided. If the cumulative absorbed breast dose is less than around 10 mGy and the annual breast cancer mortality reduction is 25% (as in our first scenario), the ratio between gained/lost years of life and avoided/induced breast cancer deaths will exceed 10, independent of assumption of the radiation risk. However, there are scenarios, not completely unrealistic, that could give small net benefits or no benefit at all. In the Swedish data on routine mammography the absorbed breast dose per view was between 0.7 and 3.2 mGy at the various screening centres (W Leitz, unpublished data). Furthermore, the values for ERR $Gy^{-1}$ in the cohort studies referred differ within a factor of four (Mattsson et al, 1995). So, considering a screening programme with annual, two-view examinations with an absorbed dose per view of 3 mGy, giving a total dose of ≈50 mGy per invited woman and assuming ERR $Gy^{-1}$ to be the value observed in the benign breast disease study (Mattsson et al, 1995), the net benefit will be reduced considerably. If the reduction in annual breast cancer mortality is less than 20% there is no net benefit for avoided breast cancer deaths (Figures 4 and 5). It is also noteworthy to point out that the number of radiation-induced cases in this scenario is only contributing to a relatively small increase of the cumulative incidence of breast cancer (0.2 percentage units for a follow-up to 100 years of age).

This study has focused on the risk of radiation induced breast cancer and this will influence the positive effect of screening in terms such as gained years of life for a follow-up throughout life. It is important that the follow-up is covering the whole lifetime, because accessible data mainly reflects early positive effects (10–15 years after screening start) whereas radiation-induced breast cancer deaths are showing up after a considerably longer period of time. With reasonable assumptions (such as in our scenarios) the group 40–49 years of age will get a relatively large benefit from mammographic screening in terms of years of life gained or breast cancer deaths avoided.

A validation of mammographic screening programmes must include also other factors like psychological effects and costs. In normal circumstances the radiation risk is not crucial in the overall assessment of the outcome of the programme. However, as shown in this paper, the margins for women below age 50 are not very large. When losing control over the radiation doses involved there is a tangible risk that the net benefit will be reduced to a questionable low value or even be turned into its opposite.

*© 2000 Cancer Research Campaign*

### ACKNOWLEDGEMENTS

This study was supported by the Cancer Society in Stockholm. We thank Director-General Lars-Erik Holm, Associated Professor Sven Törnberg, Professor Arne Wallgren, Mr Hemming Johansson for fruitful discussions and valuable comments on the manuscript, and Mrs Elisabeth Bjurstedt for skillful assistance.

### REFERENCES

Andersson I and Janzon L (1997) Reduced breast cancer mortality in women under age 50: updated results from the Malmo Mammographic Screening Program. *J Natl Cancer Inst Monogr* **22**: 63–67

Berrino F, Sant M, Verdecchia A, Capocaccio R, Hakulinen T and Estève J (1995) *Survival of Cancer Patients in Europe*. IARC: Scientific Publications 132. IARC: Lyon

Bjurstam N, Bjorneld L, Duffy SW, Smith TC, Cahlin E, Eriksson O, Hafstrom LO, Lingaas H, Mattsson J, Persson S, Rudenstam CM and Save-Soderbergh J (1997) The Gothenburg breast screening trial: first results on mortality, incidence, and mode of detection for women ages 39–49 years at randomization. *Cancer* **80**: 2091–2094

Boice JD, Jr, Preston D, Davis FG and Monson RR (1991) Frequent chest X-ray fluoroscopy and breast cancer incidence among tuberculosis patients in Massachusetts. *Radiat Res* **125**: 214–222.

Fletcher SW, Black W, Harris R, Rimer BK and Shapiro S (1993) Report of the International Workshop on Screening for Breast Cancer. *J Natl Cancer Inst* **85**: 1644–1656.

Gordis L, Berry DA, Chu SY, Fajardo LL, Hoel DG, Laufman LR et al (1997) National Institutes of Health Consensus Development Conference Statement: breast cancer screening for women ages 40–49, January 21–23, 1997. *J Natl Cancer Inst* **89**: 1015–1026.

IARC (1992) *Cancer Incidence in Five Continents*, Vol. VI. International Agency for Research on Cancer: Lyon.

Land CE (1980) Estimating cancer risks from low doses of ionizing radiation. *Science* **209**: 1197–1203.

Mattsson A, Ruden BI, Palmgren J and Rutqvist LE (1995) Dose- and time-response for breast cancer risk after radiation therapy for benign breast disease. *Br J Cancer* **72**: 1054–1061.

NIH (1985) *Report of the National Institutes of Health ad hoc Working Group to Develop Radioepidemiological Tables*. NIH Publication No. 85-2748. Office of the Director, National Institutes of Health: Washington, DC.

Nystrom L, Rutqvist LE, Wall S, Lindgren A, Lindqvist M, Ryden S, Andersson I, Bjurstam N, Fagerberg G, Frisell J and et al (1993) Breast cancer screening with mammography: overview of Swedish randomised trials. *Lancet* **341**, 973–978.

Rimer BK (1996) Breast cancer screening. In: *Diseases of the Breast*, Harris JR, Lippman ME, Morrow M and Hellman S (eds) pp. 307–322. Lippincott-Raven: Philadelphia

Shapiro S, Venet W, Strax P and Venet L (1988) Current results of the breast cancer screening randomized trial: the Health Insurance Plan (HIP) of Greater New York Study. In: *Screening for Breast Cancer*, Day N and Miller A (eds), pp. 3–15. Hans Huber: Toronto

Shore RE, Hildreth N, Woodard E, Dvoretsky P, Hempelmann L and Pasternack B (1986) Breast cancer among women given X-ray therapy for acute postpartum mastitis. *J Natl Cancer Inst* **77**: 689–696.

Stockholm Breast Cancer Study Group (1997) *Breast Cancer: Diagnosis, Treatment and Follow-up in the Stockholm-Gotland Region*. Onkologic Centre, Karolinska Hospital: Stockholm (in Swedish).

Tabar L, Larsson L-G, Andersson I, Duffy SW, Nyström L and Rutqvist LE (1996) Breast-cancer screening with mammography in women aged 40–49 years. *International Journal of Cancer* **68**: 663–699.

Tokunaga M, Land CE, Tokuoka S, Nishimori I, Soda M and Akiba S (1994) Incidence of female breast cancer among atomic bomb survivors, 1950–1985. *Radiat Res* **138**: 209–23.

UNSCEAR (1988) *United Nations Scientific Committee on the Effects of Atomic Radiation. Sources, Effects and Risks of Ionizing Radiation. 1988 Report to the General Assembly, with scientific annexes*. United Nations: New York.

UNSCEAR (1993) *United Nations Scientific Committee on the Effects of Atomic Radiation. Sources and Effects of Ionizing Radiation. UNSCEAR 1993 Report to the General Assembly, with scientific annexes*. United Nations: New York.

UNSCEAR (1994) *United Nations Scientific Committee on the Effects of Atomic Radiation. Sources and Effects of Ionizing Radiation. UNSCEAR 1994 Report to the General Assembly, with scientific annexes*. United Nations: New York.

### APPENDIX

### Formulas

Subsections correspond to subsections in Material and Methods.

### Total background breast cancer mortality

$$\mathrm{BCM} = \sum_{I=40}^{100} R_B(I) \cdot L_Q(I) \sum_{J=I}^{100} S_A(J-I) \cdot D_A(J-I) \quad (1)$$

where

$R_B(I)$ = background risk of first primary breast cancer at age I years.

$L_Q(I)$ = $L_Q(I-1).\ [1-Q(I-1)]$; size of population; $L_Q(40) = 100\,000$.

$Q(I)$ = death risks. From female population of Stockholm 1980–1989 (INREGIA).

$S_A\ (J-I)$ = cumulative survival rates of breast cancer patients.

$D_A\ (J-I)$ = annual death rates of breast cancer patients.

A = different survival and deaths rates for patients of ages at diagnosis of breast cancer < 50 years, 50–69 years, and ≥ 70 years.

### Effects of mammographic screening

*Reduction in background breast cancer mortality*

$$\mathrm{RBCM} = \sum_{I=40}^{E-1} \sum_{J=40}^{100} \mathrm{BCM}(I, J-I) + \sum_{I=E}^{E+Z-1} \sum_{J=E}^{E+Z-1} \mathrm{BCM}(I, J-I) +$$

$$(1-P)\sum_{I=E}^{EL} \sum_{J=E+Z}^{100} \mathrm{BCM}(I, J-1) + \sum_{I=EL+1}^{100} LT(I) \sum_{J=EL+1}^{100} \mathrm{BCM}(I, J-I) \quad (2)$$

where

E = age in years at first screening.

Z = number of years after first screening at age E years the effect shows up.

EL = age at last screening.

P = annual relative reduction in breast cancer mortality. If different reduction levels in breast cancer mortality was assumed, e.g. for 40–49 years of age and 50–69 years of age at screening, the third term was separated in two parts accordingly.

LT = corresponds to a shift in incidence function reflecting lead time. This shift was transferred to a lower breast cancer mortality than otherwise. LT was expected to be < 1 for 3 years after last screening corresponding to mean lead time. Thereafter LT = 1.

$$\mathrm{BCM}(I,J\text{–}I) = R_B(I) \cdot L_Q(I) \cdot S_A\ (J-I) \cdot D_A\ (J-I) \text{ from } (1).$$

*Radiation-induced breast cancer cases*

$$N = \sum_{J=E,Y}^{EL} \mathrm{ERR}_D(J) \sum_{I=J}^{100} G(I-J) \cdot R_B(I) \cdot L_{SP}(I) \quad (3)$$

where

Y = step in summation, corresponded to the time interval between examinations.

© 2000 Cancer Research Campaign

$ERR_D$ (J) = $0.69D \cdot e^{-0.054D} \cdot e^{-0.06(J-40)}$ [Benign breast disease study (Mattsson et al, 1995)] or

$ERR_D$ (J) = $0.708D \cdot e^{-0.0744(J-20)}$ [Massachusetts fluoroscopy study (Boice et al, 1991)].

G(I−J) = Graded impact of $ERR_D$ (J) I–J years after exposure (NIH, 1985);

G(I−J<5) = 0;

G(5) = 0.074;

G(6) = 0.259;

G(7) = 0.50;

G(8) = 0.741;

G(9) = 0.926;

G(I−J>9) = 1.

$L_{SP}(I) = L_{SP}(I-1)\,[1-Q_O(I-1) + Q_{BC}(I-1) - R_B(I-1) - R_N(I-1)]$

= size of the screened population.

$L_{SP}$ (40) = 100 000.

$Q_O$ (I−1) = overall deaths risk;

$Q_{BC}$ (I−1) = proportion of women that die from breast cancer; added to avoid double counting.

$R_B$ (I−1) = background risk of first primary breast cancer. Women who have contracted breast cancer should not be in question for routine screening and are therefore subtracted.

$R_N$ (I−1) = radiation induced risk of breast cancer

= $N(I-1)/L_{SP}(I-1)$. The corresponding cases were subtracted with the same motivation as for $R_B$.

### *Radiation-induced breast cancer deaths*

- Assuming no effect of screening

This formula for the total number of induced breast cancer deaths is analogous to formula (1):

$$IM = \sum_{I=E}^{100} N(I) \sum_{J=I}^{100} S_A(J-I) \cdot D_A(J-I) \quad (4)$$

where

$$N(I) = R_B(I) \cdot L_{SP}(I) \sum_{J=E,Y}^{\min(EL,I)} ERR_D(J) \cdot G(I-J) \quad (5)$$

= total number of induced breast cancer cases predicted to be diagnosed a specific year of attained age. The summation over J gives the total ERR at a specific attained age associated with the cumulative exposure to ionizing radiation during the screening programme.

- Assuming effect of mammographic screening (RIM) This formula for the reduced number of radiation induced breast cancer deaths is analogous to formula (2):

$$RIM = \sum_{I=E}^{E+Z-1} \sum_{J=E}^{E+Z-1} IM(I, J-I) + (1-P) \sum_{I=E}^{EL} \sum_{J=E+Z}^{100} IM(I, J-I) + \sum_{I=EL+1}^{100} LT(I) \sum_{J=EL+1}^{100} IM(I, J-I) \quad (6)$$

where

IM(I,J−I) = $N(I) \cdot S_A(J-I) \cdot D_A(J-I)$ from (4).

### *Net effects*

- Net total number of avoided breast cancer deaths
- Net number of gained years of life

$$\sum_{I=40}^{100} [BCM(J) - RBCM(J) + RIM(J)] \quad (7)$$

$$\sum_{I=40}^{100} [\{BCM(J) - RBCM(J) + RIM(J)\}YR(J)]$$

where

YR(J) = expected number of remaining years of life at age J years.

BCM(J) = appropriate summation over I for a given attained age J years in formula 1.

RBCM (J)= appropriate summation over I for a given attained age J years in formula 2.

RIM(J) = appropriate summation over I for a given attained age J years in formula 6.

 *© 2000 Cancer Research Campaign*
